# Analysis of Spleen Cells in Susceptible and Resistant Mice with Experimental Lagochilascariosis

**DOI:** 10.5402/2013/180652

**Published:** 2012-09-12

**Authors:** Priscila Guirão Lara, Mariana Felix de Souza Prudente, Neusa Mariana Costa Dias, Denise Vilarinho Tambourgi, Ruy de Souza Lino-Junior, Mônica Spadafora-Ferreira, Mara Silvia Carvalhaes

**Affiliations:** ^1^Laboratory of Immunochemistry, Butantan Institute, São Paulo, SP, Brazil; ^2^Laboratory of Immunogenetics, Butantan Institute, São Paulo, SP, Brazil; ^3^Department of Microbiology, Immunology, Parasitology and Pathology, Institute of Tropical Pathology and Public Health, Federal University of Goiás, Goiânia, GO, Brazil

## Abstract

Lagochilascariosis is an emerging parasitic disease caused by the helminth *Lagochilascaris minor*. The experimental mouse model has been used to study the immune response against *L. minor* infection. In the present work, immunohistochemistry analysis of spleen cells populations was evaluated in susceptible (C57BL/6) and resistant (BALB/c) mice experimentally infected with *L. minor*. The BALB/c mice exhibited increased spleen cell indexes as follows: F4/80+ at 100 days after infection (DPI), CD4+ at 100 and 250 DPI, CD8+ at 35 and 100 DPI, and CD19+ at 100, 150, and 250 DPI. In the spleens of the infected C57BL/6 mice, increased indexes of the following spleen cells were observed: F4/80+ cells at 250 DPI, CD4+ cells at 150 DPI, CD8+ cells at 35, 150, and 250 DPI, and CD19+ cells at 150 to 250 DPI. The index of spleen cells confirmed the differences between the control and infected groups at several time points following the infection. These data demonstrate an association between a preferential increase in the number of CD4+ and CD19+ spleen cells and resistance to experimental lagochilascariosis in BALB/c mice and between a preferential increase in the number of CD8+ spleen cells and susceptibility in C57BL/6 mice.

## 1. Introduction

Lagochilascariosis is caused by *Lagochilascaris minor*, which can be the aetiological agent of the human infection; eventually,  it is also found in domestic animals, such as dogs and cats [1]. Although it does not constitute a public health problem, lagochilascariosis is an emerging disease in Brazil; 90% of the described cases worldwide occur in this country [2].

Lagochilascariosis is a chronic disease characterised by the presence of granulomatous lesions in the oronasopharyngeal region that cause exudative abscesses with the presence of eggs, larvae, and adult parasites and indicate the occurrence of an autoinfection. This infection can be fatal if the parasite invades the lungs and the central nervous system [3, 4].

The natural life cycle and mechanism of infection of *L. minor* are still unclear because little is known about the biology of this parasite. Wild animals, such as dogs, felines, and rodents, are considered to be likely natural reservoirs of *L. minor* [2, 5]. The experimental heteroxenic life cycle of the parasite was described using mice as intermediate hosts and domestic cats as definitive hosts [6, 7].

The extraordinary capacity of *L. minor* to migrate to different human tissues has also been observed in experimental models [8]. In mice inoculated with infective parasite eggs containing third-stage larvae (L3), hatched larvae were observed during migration to the intestinal tract. A dissemination of these larvae to other organs, such as the lungs, skeletal muscles, and subcutaneous tissue, was observed. When cats were fed the carcasses of infected mice, the L3 larvae hatched from the stomach cysts and ascended to the oropharynx, where fourth-stage larvae (L4) were found. *L. minor *eggs were found in the faeces of infected cats [9].

The host-parasite relationship during experimental infection with *L. minor* has been studied in strains of inbred mice with different genetic backgrounds. In previous studies, after observing the cumulative mortality rate within one year of infection, it was possible to classify the mouse strains as resistant (A/J, BALB.xid, and BALB/c) or susceptible (C57BL/6 and B10.A). The susceptible strains displayed reduced survival, more intense granulomatous lesions, and higher numbers of L3 larvae and adult parasites [8, 10].

It is evident that the difference in the susceptibility of mice to experimental lagochilascariosis can be influenced by the individual immune response, which plays an essential role in the progression of the infection by reducing or blocking the pathological processes [8, 11].

We have previously demonstrated that the serum levels of interleukin-10 (IL-10) were similar in *L. minor*-infected C57BL/6 and BALB/c mice at 90 DPI; however, the sera from the BALB/c mice contained higher levels of interferon-*γ* (IFN-*γ*) than those from C57BL/6 mice. During this period, less severe pulmonary lesions and fewer subcutaneous nodules containing L3 larvae were observed in BALB/c mice than in C57BL/6 mice [12]. Moreover, we have recently demonstrated that both strains produce different classes of antibodies in response to crude extract and soluble antigens from the parasite [11, 13].

Based on these previous results, we considered it important to evaluate the populations of spleen cells during experimental infection with *L. minor*. Because the profiles of spleen cells during experimental lagochilascariosis have never been studied, we accomplished the immunophenotyping of F4/80+, CD4+, CD8+, and CD19+ cells in the spleens of control mice and BALB/c and C57BL/6 mice that were experimentally infected with *L. minor*; the goal of this study was to identify alterations in the cellular populations during experimental lagochilascariosis infection.

## 2. Materials and Methods 

### 2.1. Parasite Eggs

Parasite eggs were isolated from the faeces of *Felis domesticus* individually fed the carcasses of mice infected with 30 to 100 nodules of infective third-stage larvae (L3) from a human *L. minor *isolate. The faeces of infected cats were subjected to Hoffman's method of sedimentation and stored in a formalin solution (1%) at room temperature for 40 days [14]. After the infective eggs containing L3 developed, faecal samples were subjected to Faust's method to optimally recover eggs that were free of faecal debris [15]. The egg suspensions were washed five times (20 minutes/4000 rpm) with phosphate-buffered saline (PBS, pH 7.4) and transferred to a graduated centrifuge tube. The eggs were counted on microscope slides, and the final concentration was adjusted to 10^4^ eggs/mL and used to inoculate the mice [16].

### 2.2. Mice and Experimental Infection

Mice were obtained from the Butantan Institute animal facilities. The animals were given food and water ad libitum and handled according to the local regulations. The research protocols were approved by the Ethics Committees of the Federal University of Goiás and the Butantan Institute.

A total of 20 BALB/c and 20 C57BL/6 mice were orally inoculated with a suspension of 10^3^ ± 200  *L. minor* eggs per animal. Groups of five animals were sacrificed at each time point (35, 100, 150, and 250 DPI) and submitted to necropsy. The spleens were harvested for immunohistochemistry and analysis of the cell populations. A total of 20 BALB/c and 20 C57BL/6 mice received saline orally and were used as uninfected controls at the same time points.

### 2.3. Immunohistochemistry

The mouse spleens were collected, and fragments were put into a mould and covered with OCT, an inclusion tissue compound. The fragments were then immersed in cooled isopentane (Vetec, Brazil), snap-frozen in liquid nitrogen, and stored at −80°C until use. The fragments were sectioned with a cryostat, and the tissue sections were fixed in cold acetone for 10 minutes; they were then stored at −80°C. The sections were subjected to peroxidase blocking with 30 volumes of H_2_O_2_ diluted 1/1000 in methanol (15 minutes) and then incubated with normal goat serum (15 minutes) to block nonspecific binding. After the excess serum was removed, the sections were incubated with primary rat anti-mouse monoclonal antibodies (mAb) diluted in PBS containing 2% foetal bovine serum in a humid chamber for 18 h at 4°C. The following rat anti-mouse mAb were used: anti-F4/80 (macrophage), clone A3-1 (Serotec); anti-CD19 (B lymphocytes), clone 1D3 (BD Biosciences); anti-CD4 (T-helper lymphocytes), clone H129.19 (BD Biosciences); and anti-CD8 (cytolytic T lymphocytes), clone 53-6.7 (BD Biosciences). The slides were washed in PBS and incubated with a secondary biotinylated anti-rat IgG (BD Biosciences) for 45 min. After another wash in PBS was performed, avidin-peroxidase was added for 30 minutes (room temperature), and the slides were washed again in PBS. Subsequently, the slides were stained with a diaminobenzidine substrate solution for 3 minutes and counterstained in Mayer's hematoxylin solution (Merck, Germany).

### 2.4. Quantification of Spleen Cell Subpopulations

The fields for quantification of splenic cells were imaged using a camera (Cyber shot DSC-S85) coupled to a microscope and a computer for digitisation. The cell populations were analysed using the Image J software program (NIH-EUA). The positively stained cells were quantified, in the 30 crossings of the grating, in each of 30 analysed fields. 

The accumulated median was calculated [17], and the results were presented as the median and medium deviation. The index of the stained cells (CIs) was calculated as the ratio of the mean number of positively stained cells in the infected animals versus the mean number of positively stained cells in the uninfected control animals in the different groups.

### 2.5. Statistical Analysis

The statistical analysis was performed with the GraphPad Prism 4.0 software program. All of the variables were tested for normal distribution and homogeneous variance. The means and standard deviations were used for the analysis of one variable followed by the Mann-Whitney test, and the ANOVAs were followed by Tukey's test for multiple comparisons. The differences were considered significant when *P* < 0.05.

## 3. Results

**Figure 1:**
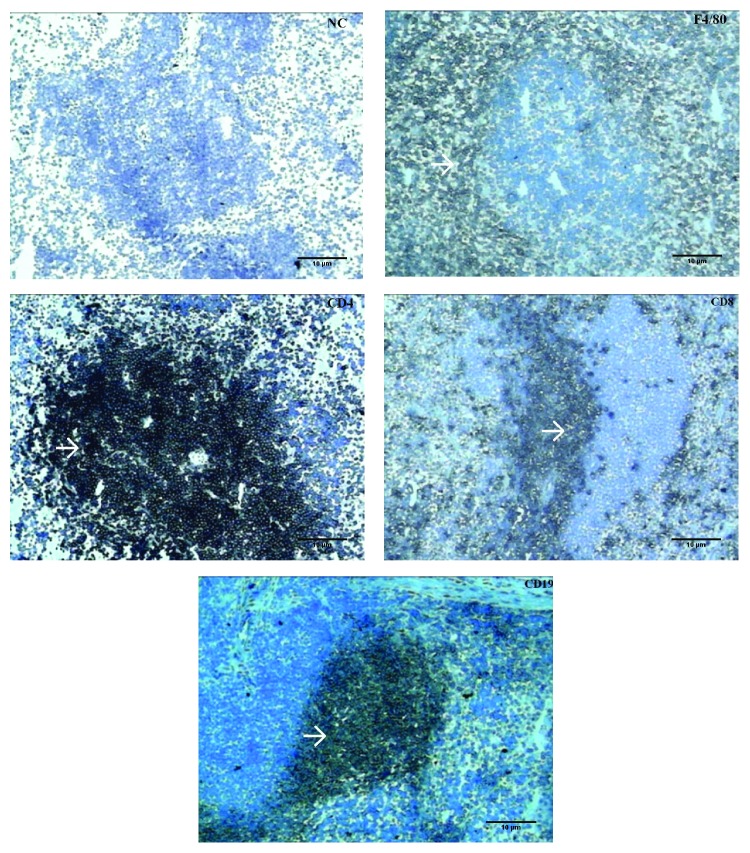
Immunohistochemistry of the spleens of infected BALB/c mice, without the demarcation antibody (NC, negative control) and with the antibodies specific for F4/80, CD4, CD8, and CD19. The arrows indicate cellular demarcation in the follicular area of the white pulp of the spleen, 100x magnification.

The spleen immunohistochemical analysis revealed a predominance of F4/80+ cells in the red pulp and a predominance of CD4+, CD8+, and CD19+ cells in the white pulp (Figure 1).

**Figure 2:**
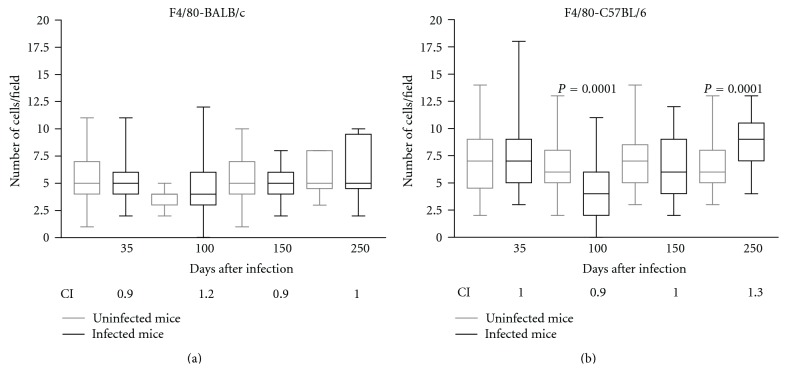
Immunohistochemical results demonstrating the presence of F4/80+ cells in the spleens of noninfected BALB/c (a) and C57BL/6 (b) mice (controls—gray line) or in those infected orally with 1000 *Lagochilascaris minor* eggs (infected—black line) at different time points following the infection. The results are expressed as the median number of cells per field; the columns indicate the standard deviation and the bars indicate the minimum and maximum values of the marked cells (∗*P* < 0.05, Mann-Whitney test). CI: index of F4/80+ cells, determined by dividing the arithmetic average of the number of marked cells per field in the infected animals by the arithmetic average of the number of marked cells in the noninfected control mice at various time points after infection.

The BALB/c animals did not present a significant variation in the number of F4/80+ cells during the course of infection (Figure 2(a)). On the other hand, the infected C57BL/6 animals displayed reduced numbers of F4/80+ spleen cells at 100 DPI (*P* < 0.01) and increased numbers of these cells at 250 DPI (*P* < 0.01) compared to the controls (Figure 2(b)). However, we detected an increased CI of F4/80+ spleen cells in the BALB/c mice at 100 DPI (CI = 1.2) and in the C57BL/6 animals at 250 DPI (CI = 1.4) (Figures 2(a) and 2(b)). The CI of the stained cells was consistent with the results that were obtained from the median but also revealed some important differences among some statistically insignificant differences among samples.

**Figure 3:**
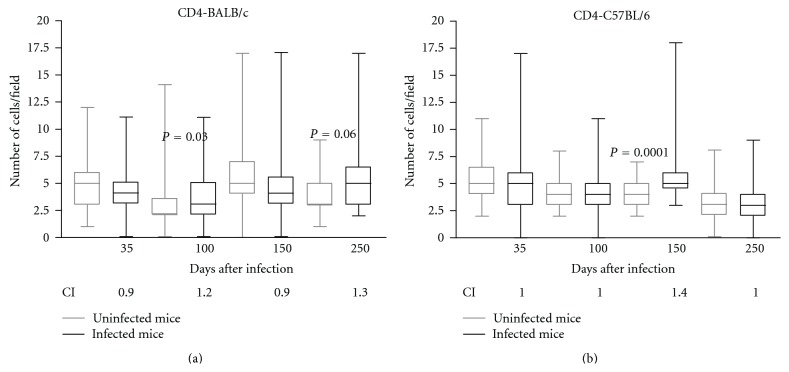
Immunohistochemical results demonstrating the presence of CD4+ cells in the spleens of noninfected BALB/c (a) and C57BL/6 (b) mice (controls—gray line) or in those infected orally with 1000 *Lagochilascaris minor* eggs (infected—black line) at different days following the infection. The results are expressed as the median number of cells per field; the standard deviations are indicated by columns, and the minimum and maximum values are indicated by bars (∗*P* < 0.05, Mann-Whitney test). CI: index of CD4+ cells, determined by dividing the arithmetic average of the number of marked cells per field in the infected animals by the arithmetic average of the number of marked cells in the noninfected control mice at different days following infection.

The number of CD4+ spleen cells increased in the infected BALB/c mice compared to the control mice at 100 DPI (*P* < 0.05) (Figure 3(a)); in the C57BL/6 mice, increased numbers of CD4+ cells were observed at 150 DPI (*P* < 0.05) (Figure 3(b)). We detected an increased CI of the CD4+ spleen cells in the BALB/c mice at 100 (CI = 1.2) and 250 (CI = 1.3) DPI and in the C57BL/6 animals at 150 DPI (CI = 1.4) (Figures 3(a) and 3(b)). Again, the results obtained from the CI of the stained cells were consistent with those obtained by the median but also showed some important differences among samples that were not statistically significant.

**Figure 4:**
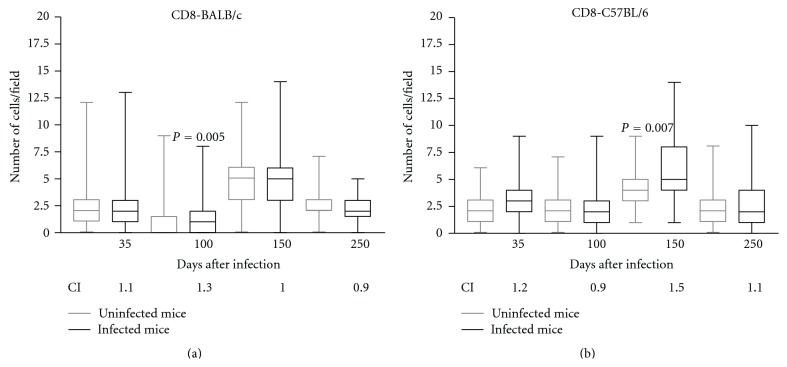
Immunohistochemical results demonstrating the presence of CD8+ cells in the spleens of noninfected BALB/c (a) and C57BL/6 (b) mice (controls—gray line) or in those infected orally with 1000 *Lagochilascaris minor* eggs (infected—black line) at different days following infection. The results are expressed as the median number of cells per field; the standard deviations are indicated by columns, and the minimum and maximum values are indicated by bars (∗*P* < 0.05, Mann-Whitney test). CI: index of CD8+ cells, determined by dividing the arithmetic average of the number of marked cells per field in the infected animals by the arithmetic average of the number of marked cells in the noninfected control mice at different days following infection.

The number of CD8+ cells in the spleens of infected BALB/c mice was significantly increased only at 100 DPI when compared with the controls (*P* < 0.05) (Figure 4(a)). In the infected C57BL/6 mice, the number of CD8+ cells increased in comparison with the controls at 150 DPI (*P* < 0.05; Figure 4(b)). The CI of the CD8+ cells in the spleen increased at 35 (CI = 1.1) and 100 DPI (CI = 1.3) in the BALB/c mice and at 35 (CI = 1.2), 150 (CI = 1.5), and 250 (CI = 1.1) DPI in the C57BL/6 mice (Figures 4(a) and 4(b)). Again, the CI of the stained cells are consistent with the results that were obtained by the median but also reveals some important differences among samples that are not statistically significant.

**Figure 5:**
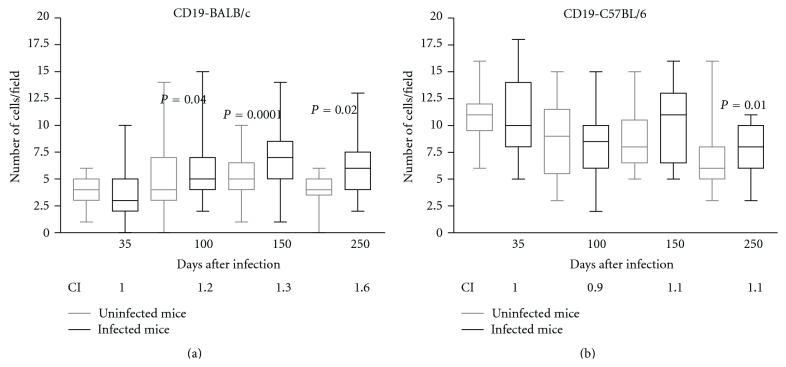
Immunohistochemical results demonstrating the presence of CD19+ cells in the spleens of noninfected BALB/c (a) and C57BL/6 (b) mice (controls—gray line) or in those infected orally with 1000 *Lagochilascaris minor* eggs (infected—black line) at different days following infection. The results are expressed as the median number of cells per field; the standard deviations are indicated by columns, and the minimum and maximum values are indicated by bars (∗*P* < 0.05, Mann-Whitney test). CI: index of CD19+ cells, determined by dividing the arithmetic average of the number of marked cells per field in the infected animals by the arithmetic average of the number of marked cells in the noninfected control mice at different days following infection.

The number of CD19+ cells in the spleens was increased at 100, 150, and 250 DPI (*P* < 0.05) in the infected BALB/c mice in comparison with the controls (Figure 5(a)); in the C57BL/6 mice, the number of CD19+ cells increased significantly (*P* < 0.05) at 250 DPI (Figure 5(b)). The spleen CI of the CD19+ cells increased at 100 (CI = 1.2), 150 (CI = 1.3), and 250 DPI (CI = 1.6) in the BALB/c mice and at 150 (CI = 1.1) and 250 DPI (CI = 1.1) in the C57BL/6 mice (Figures 5(a) and 5(b)). The CI of the stained cells is consistent with the results obtained by the median and shows some important differences among samples that are not statistically significant. 

## 4. Discussion 

The number of F4/80+ cells in the spleens of infected mice increased significantly at 250 DPI in the C57BL/6 mice. The index of the F4/80+ cells in the spleens of the infected mice increased at 100 DPI in the BALB/c mice and at 250 DPI in the C57BL/6 mice. The macrophages of the red pulp were F4/80+, and their role is to remove antigens and red blood cells. Certain subsets of mature macrophages residing in the T-cells areas of the spleen express low levels of F4/80. We should consider that resident macrophages with different phenotypic and functional characteristics, and their precursors in the blood, the monocytes that infiltrate the tissues, can be answering to the helminth in the infection site and the expression of F4/80 can vary during the activation and macrophage maturation [18, 19]. During experimental infection with *L. minor*, F4/80+ cells can play an important role in the orchestration of the immune response by producing proinflammatory cytokines with neutrophil-activating potential, regulatory cytokines that modulate the inflammation, and cytokines that aid in the differentiation of CD4+ lymphocyte subsets. 

In mice spleens, a macrophage population characterised by an F4/80(high) Mac-1(low) phenotype produces suppressor cytokines, such as TGF-*β* and IL-10 [20]. Some studies have demonstrated that alternatively activated macrophages (AAMs) are associated with resistance to parasitic infections and the production of cytokines and angiogenic growth factors during helminth infections [21]. Classically activated macrophages (CAMs) that produce nitric oxide (NO), which is cytotoxic to metazoan larvae [22], also seem to be important in helminthosis resistance [23]. 

The BALB/c mice exhibited an increased CI of the CD4+ spleen cells at 100 and 250 DPI, and such an increase was observed in the C57BL/6 mice only at 150 DPI. It has been suggested that these CD4+ cells are resident in the spleen or are lymphocytes that have migrated from the blood to the marginal zone (MZ) of the spleen [24]. The modulation of different CD4+ lymphocyte subsets has been observed in many experimental models of helminthosis [25]. The fact that higher levels of IFN-*γ* are found in the serum of BALB/c mice than in that of C57BL/6 mice suggests the participation of cells with a Th1 profile in resistance to infection [12]. Several studies have described the participation of Th17 cells in the aggravation of the pathology caused by schistosomiasis [26–28]; in fact, the presence of neutrophils in the granulomatous lesions that was observed in the C57BL/6 mice suggests the participation of these Th17 cells in this strain [12]. 

Our results demonstrated an increase in the CI of the CD8+ spleen cells in the C57BL/6 mice at 35, 150, and 250 DPI. The susceptibility of mice chronically infected with *Trichuris muris* is associated with high numbers of CD8+ T cells [29]. The CD8 molecule can be expressed in the CD8*αα* configuration in T*γδ* lymphocytes [30], and these cells have been described in other cases of helminthosis [31]. It is necessary to further evaluate the participation of CD8+ noncytolytic lymphocytes that produce inflammatory and regulatory cytokines during the course of *L. minor *infection. The preferential increase of these cells in C57BL/6 mice is apparently associated with the susceptibility to experimental infection. 

The BALB/c mice displayed increased indexes of CD19+ spleen cells at 100, 150, and 250 DPI. The CD19+ molecule is present at the surface of practically all B lymphocytes; it operates as a coreceptor to amplify the BCR signal cascade [32]. B lymphocytes possess a variety of immune functions that contribute to protective immunity during helminth infections, including the production of antibodies and cytokines, and the presentation of antigens [33]. During experimental infection with *Heligmosomoides polygyrus*, antibodies block the intestinal invasion in the earliest days of the infection [34]. BALB/c mice have been demonstrated to produce IgM, IgG, IgA, and IgE against the parasite's crude extract (CE) and secreted-excreted (SE) antigens, with a higher production of IgA, IgM, and IgG in response to the CE antigens than to the SE products of *L. minor* [11]. These results suggest that the large number of CD19+ cells in the spleens of BALB/c mice is associated with the increased production of antibodies that participate in the resistance to *L. minor* infection. 

At 100 DPI, an increased CI of the F4/80+, CD4+, CD8+, and CD19+ spleen cells was demonstrated in the BALB/c mice. At this time point, we observed less intense lung lesions and fewer subcutaneous encysted L3 larvae in the BALB/c strain compared to the infected C57BL/6 strain. 

The information gained from the immunohistochemistry regarding the mean, standard deviation, and the CI of the stained cells demonstrated an association between the preferential increase in the number of CD4+ and CD19+ spleen cells with resistance to experimental lagochilascariosis in the BALB/c mice and between the preferential increase of CD8+ spleen cells and susceptibility in the C57BL/6 mice.
